# Fe and As geochemical self-removal dynamics in mineral waters: evidence from the Ferrarelle groundwater system (Riardo Plain, Southern Italy)

**DOI:** 10.1007/s10653-021-00891-5

**Published:** 2021-04-09

**Authors:** Emilio Cuoco, Stefano Viaroli, Vittorio Paolucci, Roberto Mazza, Dario Tedesco

**Affiliations:** 1 Istituto Nazionale Di Geofisica E Vulcanologia, Osservatorio Vesuviano, Via Diocleziano 328, 80124 Napoli, Italy; 2 Dipartimento Di Scienze, Università degli Studi Roma Tre, Largo S. Leonardo Murialdo 1, 00146 Roma, Italy; 3 Ferrarelle S.p.A., Contrada Ferrarelle, Riardo, Italy; 4 Dipartimento Di Scienze E Tecnologie Ambientali, Biologiche E Farmaceutiche, Università Della Campania “L. Vanvitelli”, Via Vivaldi 43, 81100 Caserta, Italy

**Keywords:** Fe and As hydrogeochemistry, Natural mineral water, Water treatment, Oxohydroxides adsorption, Natural self-removal dynamics

## Abstract

A theoretical pattern for Fe and As co-precipitation was tested directly in a groundwater natural system. Several monitoring wells were sampled to identify the different endmembers that govern the hydrodynamics of the Ferrarelle Groundwater System in the Riardo Plain (Southern Italy). In agreement with recent investigations, we found a mix of a deep and a shallow component in different proportions, resulting in a specific chemical composition of groundwater in each well depending on the percentages of each component. The shallow component was characterized by EC ~ 430 µS/cm, Eh ~ 300 mV, Fe ~ 0.06 µmol/L and As ~ 0.01–0.12 µmol/L, while the deep component was characterized by EC ~ 3400 µS/cm, Eh ~ 170 mV, Fe ~ 140 µmol/L and As ~ 0.59 µmol/L. A general attenuation of As and Fe concentration that was not due to a simple dilution effect was observed in the mixing process. The oxidation of Fe(II) to Fe(III) produces solid precipitates which adsorb As from solution and then co-precipitate. The reactions pattern of Fe(II) oxidation and As adsorption gave a linear function between [As] and [Fe], where the angular coefficient depends on the [O_2_]/[H^+^] ratio. Chemical data obtained from our samples showed a very good agreement with this theoretical relationship. The investigated geochemical dynamics represented a natural process of attenuation of Fe and As, two undesirable elements that usually affect groundwater quality in volcanic aquifers in central-southern Italy, which are exploited to supply drinking water.

**Graphic abstract:**

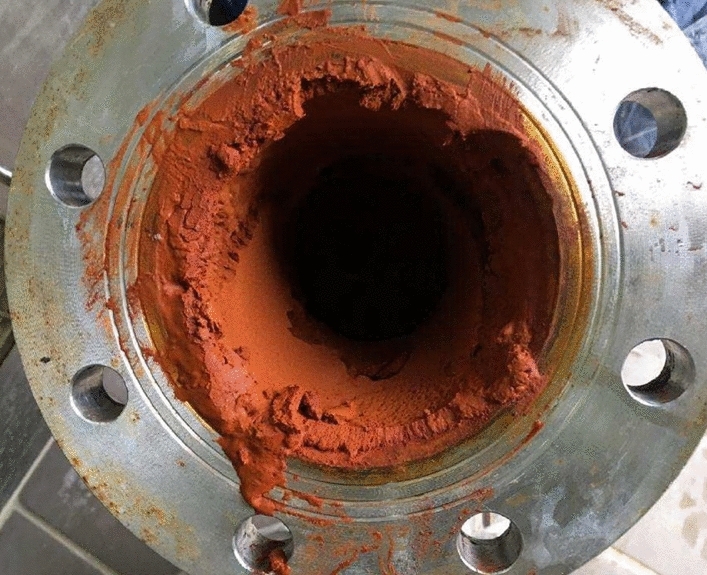

## Introduction

Fe and As are naturally present in groundwater. When the concentration of one or both of these elements exceed safety thresholds in drinking water, they represent a threat for human health (Plant et al., 2014; World Health Organization [WHO], 2017). Several studies worldwide have been dedicated to natural contamination problems, in particular of As in water resources and the effects on human health (e.g., Ahn, 2011; Ali et al., 2019; Anawar et al., 2003; Das et al., 1996; Mukherjee et al., 2014; Ravenscroft, 2009; Smedley & Kinniburgh, 2002). The As mean concentration in the earth's crust is 2 mg/Kg, variable from igneous rocks (1–4 mg/Kg) to limestone (1.4 mg/Kg) and shale (17 mg/Kg) (Tanaka, 1988). Despite the lower As contents, volcanic glasses from basaltic–andesitic magmas are often responsible for high As concentrations in waters due to their high reactivity in alteration processes (Smedley et al., 2002). In fact, the presence of As over the WHO threshold (10 µg/L = 0.13 µmol/L) (WHO, 2017) is mainly due to the amount of rock altered by groundwater interactions with As-bearing host rocks. The increase in water solutes is due to the amount of alteration of host rock and it can mainly be related to (1) the reactivity of glass/minerals in the rocks with waters, (2) the aggressiveness of water solutions (*e.g.,* acidity amount, presence of reactive gases) and (3) the residence time of water in the host rock. The combination of these three factors has generated serious worldwide episodes of As natural contamination of groundwater resources (Romero-Schmidt et al., 2001; Wickramasinghe et al., 2004; Xia et al., 2007; Heredia & Cirelli, 2009; Pokhrel et al., 2009; Chakrabarti et al., 2016; Yunus et al., 2016; Litter et al., 2019). A series of Pleistocene volcanic structures, associated with productive CO_2_ saturated aquifers can be found in central-southern Italy (peri-Tyrrhenian belt) (Cataldi, 1995; Minissale, 2004; Chiodini et al., 2013; Peccerillo, 2017). The interactions between groundwater and different effusive products (from sub-alkaline to alkaline basalts, ultrapotassic mafic to ultramafic rock-types, Serri et al., 2001) has led to important issues related to As presence in water resources (Parrone et al., 2020 and therein references). The problem of excessive As concentration is often coupled with high amounts of Fe (Gosh et al., 2020). Fe can be present in aquifers as dissolved ion (Fe^2+^) under reducing conditions and alternatively, as insoluble oxohydroxide under oxidizing conditions (Di Curzio et al., 2019; Palmucci et al., 2016; Stumm & Morgan, 1996) giving rise to precipitates and/or mobilization as colloids (Viaroli et al., 2016). The increasing oxygenation degree of Fe-rich groundwater leads to Fe(OH)_3_ precipitation due to oxidation from Fe(II) to Fe(III) (Houben, 2003). As suggested by Appelo and de Vet (2003), ferrihydrite may be representative of the iron-oxohydroxides which form during in situ iron removal in aquifers. This hypothesis seems to be confirmed by more recent investigations (e.g., Dekov et al., 2014; Hirst et al., 2020). The structure of ferrihydrite is not univocally determined at present because of the very fine particle size, the existence of poorly ordered versions of the mineral and the lack of synthetic well crystallized material (Bowles, 2021). However, this precipitate has a high reactivity and large surface area (Davis & Leckie, 1978) due to the complex surface. These characteristics make ferrihydrite a significant adsorbent of metals in natural waters. It is highly likely that the metal sorption is mainly due to singly coordinated oxygens on the ferrihydrite surface (Hiemstra & van Riemsdijk, 2009).

The iron hydroxides can adsorb As, removing it from the solution (Appelo et al., 2002; Goldberg, 1986; Hiemstra & van Riemsdijk, 1999). This chemical process is at the basis of the debate about the use of different remediation techniques against the presence of As (Ahmad et al., 2020; Hao et al., 2018). Volcanic aquifers of southern-central Italy represent strategic resources for drinking water supply, although the groundwater is often affected by high Fe and As concentrations (Angelone et al., 2008; Cinti et al., 2017; Madonia et al., 2017).

Tap water can be treated according to the current European Council 98/83/CE directive in order to limit or remove chemical or biological contaminations and to reach values in agreement with the maximum allowed concentrations. On the contrary, the 2009/54/CE directive on the exploitation and marketing of natural mineral waters limited the treatment procedures, which can be applied to natural mineral waters (NWM) like the separation of unstable elements, such as iron and sulfur compounds (SO_4_^2−^, H_2_S, HS^−^) by filtration or decanting, possibly preceded by oxygenation or the treatment with ozone-enriched air. In the EU, the natural mineral waters are treated by a filtration through oxohydroxides media according to the European Food Safety Authority (EFSA) recommendations (EFSA, 2008). It has been demonstrated that major ions of NMWs have little affinity with iron and manganese oxohydroxides and that their concentration in the NMW is not modified through treatments with these tools (Mohan & Pittman, 2007). These treatments thus meet the basic requirements of Directive 2009/54/CE, which request that the composition of the NMW is not altered as regards the essential constituents.

EFSA (2008) assessed the usable categories of media which allow the removal of arsenic, iron and manganese from NMW: (1) Iron and/or manganese coated silica sand, alumina or zeolite (obtained by self-coating when flowing water with high concentrations in iron and/or manganese on sand, on alumina or on zeolite), (2) Natural manganese ores (ground, washed and sieved) and (3) Synthetic iron-based oxohydroxides.

Nevertheless, the use of such treatments raises issues about management costs and about risk assessment related to efficacy of the removal of As and Fe, potential microbiological and chemical contamination through the treatment (EFSA, 2008). In some cases, deep and reducing groundwater mixes with the more oxygenated shallow aquifers giving rise to natural iron precipitation without any treatment. This dynamic was observed in the Ferrarelle Groundwater System located in the Riardo Plain (Southern Italy) (Cuoco et al., 2020). *Ferrarelle™* is one of the biggest and most famous Italian water bottling companies, and the exploited aquifer has recently been deeply investigated (Viaroli et al., 2016; Viaroli et al., 2018, 2019a; Cuoco et al., 2020; Sacchi et al., 2021). The achieved results revealed that the entire groundwater system is mainly governed by mixing dynamics between (a) the groundwater ascending under natural pressure from the deep aquifer, which is CO_2_ saturated (0.120 mol/Kg), highly mineralized (~ 3000 µS/cm) and reduced (Eh = 170 mV), hosted in the carbonate basement and (b) the shallow aquifer, which is CO_2_-poor (0.007 mol/Kg), weakly mineralized (~ 400 µS/cm) and has higher Eh (~ 300 mV). According to the described conditions, a decrease in As related to iron precipitation during the mixing dynamics was observed. The Ferrarelle bottled water is therefore naturally characterized by lower As and Fe content than the legal allowable concentrations. The present study investigates the pattern of chemical reactions related to Fe-oxidation and As-adsorption on formed oxohydroxides directly tested on a natural groundwater system. The outcomes give a detailed description of the geochemical self-removal dynamics of these two undesirable elements which can also be applied to other elements sensitive to the adsorption effect (e.g., Mn, U, Ba, etc.). The knowledge acquired suggests new processes that would help to improve the management of critical issues related to excessive amounts of Fe and As in water resources taking advantages from the natural geochemical processes occurring in the aquifer.

## Geological and hydrogeological settings

**Fig. 1:**
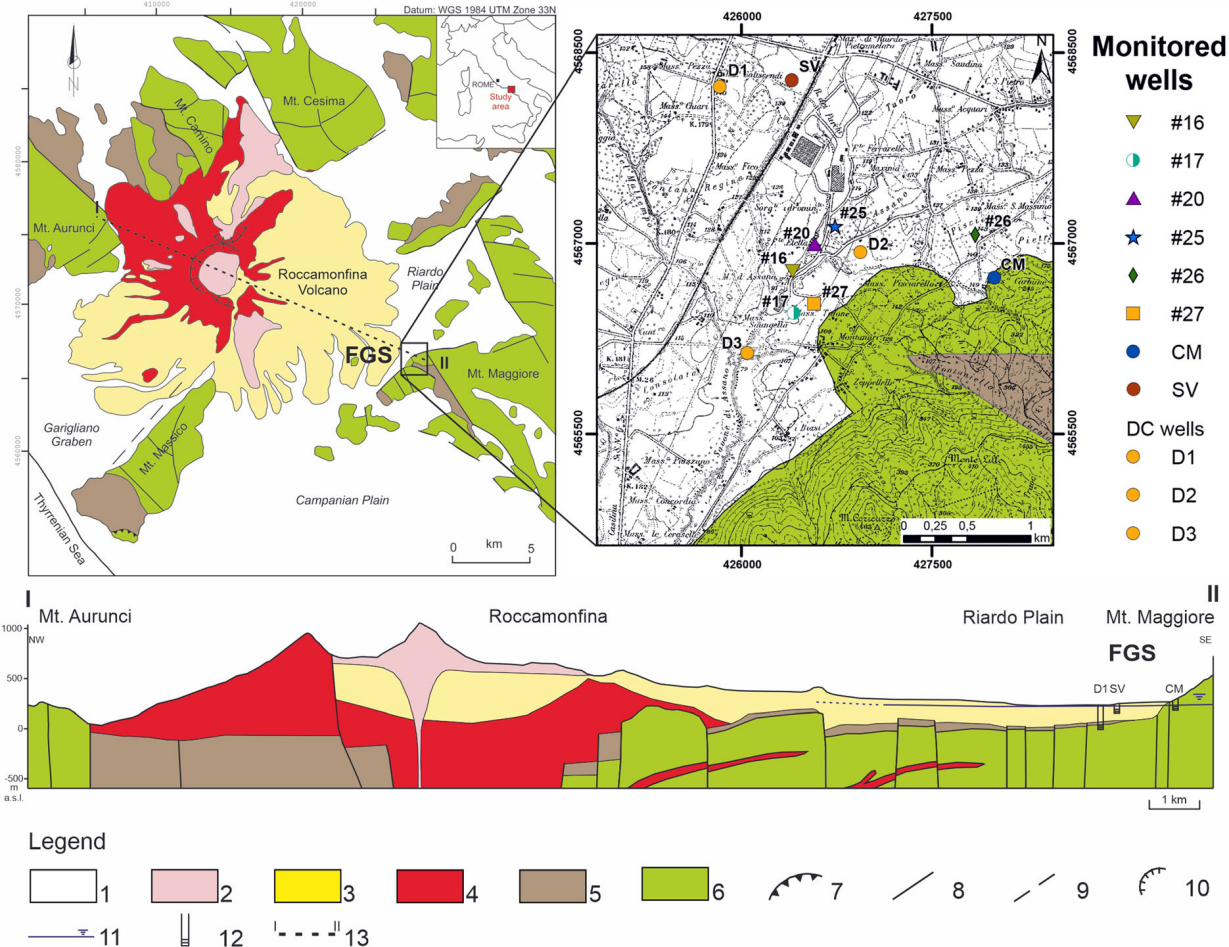
Geological map, location of the monitored wells and cross section of the study area (modified from Viaroli et al., 2019). Legend: (1) Alluvial and detritus deposits (Holocene); (2) Volcanic deposits emplaced during the 3rd phase of the Roccamonfina Volcano activity (Pleistocene); (3) Volcanic deposits emplaced during the 2nd phase of the Roccamonfina Volcano activity (Pleistocene); (4) Volcanic deposits emplaced during the 1st phase of the Roccamonfina Volcano activity (Pleistocene); (5) Synorogenic terrigenous deposits (Miocene); (6) Carbonate units (Mesozoic); (7) Thrust fault; (8) Normal and strike-slip faults involving the sedimentary basement; (9) Normal and strike-slip faults involving the quaternary units; (10) Caldera rims; (11) Water table; (12) Monitored wells; (13) Geological cross-section trace.

The Ferrarelle Groundwater System (FGS) is a multilayer aquifer, interconnected by normal faults (Cuoco et al. 2020; Viaroli et al., 2016), in the Riardo Plain between the Roccamonfina Volcano and the northern flank of Mt. Maggiore carbonate relief (Fig. 1). The carbonate formations of Mt. Maggiore and other neighboring reliefs correspond to the outcrop of the sedimentary basement (Patacca & Scandone, 2007) largely affected by a strong dislocation during the Plio-Pleistocenic extensional tectonics, producing horst-grabens structures (Cosentino et al., 2006; Giordano et al., 1995). The carbonate succession is covered by synorogenic Miocene terrigenous deposits, outcropping only in limited sectors. The activity of Roccamonfina Volcano started 550 ka BP (Rouchon et al., 2008) in correspondence with a depression formed by the cross-link of NE-SW and NW-SW oriented grabens (Capuano et al., 1992; Peccerillo, 2005). The effusive products of the first eruptive period are characterized by silica undersaturated highly potassic chemistry. This type of rocks, defined as HKS (Appleton, 1972; Peccerillo & Manetti, 1985), have significant Fe (4–8 wt%) and As (27–8 ppm) content (Conticelli et al., 2009; Rouchon et al., 2008) and mainly outcrop in the NW sector of the volcanic edifice. The aquifer hosted in these deposits has significant As content, up to 25 µg/L, whereas the Fe content is low (~ 24 µg/L) according to the oxidant conditions of the groundwater (Cuoco et al., 2010). The second phase mainly corresponds to explosive activity which started 350 ka BP (De Rita & Giordano, 1996). During this phase, undersaturated lavas and pyroclastic deposits, defined as KS-series, were emplaced (Appleton, 1972). In the Riardo Plain, only pyroclastic deposits or reworked volcanic deposits related to this volcanic phase were detected (Giordano et al., 1995), covering the sedimentary basement (Fig. 1). The volcanic activity ended with the third phase, which was mainly characterized by intracaldera activity and the emplacement of two latitic domes in the caldera summit (Giannetti, 2001). Nowadays, although the Roccamonfina volcano is extinct, CO_2_ emissions are still present on the volcanic edifice and in the surrounding plains, often coupled with soda springs, thermal and/or mineralized aquifers (Corniello et al., 2015; Cuoco et al., 2015, 2017). The FGS is CO_2_ hyper-saturated groundwater hosted in a fractured carbonate aquifer. The huge presence of both alkali and alkali earth metals, coupled with a high concentration of Fe (~ 5 mg/L) and As (up to 30 µg/L) (Cuoco et al., 2010) is proof of the lateral contribution of the basal Roccamonfina aquifer in agreement with the hydrogeological model proposed by Capelli et al., (1999). Natural pressure forces deep groundwater to flow upward along normal faults, locally mixing with the volcanic aquifer above. Chemical evidence of this mixing was discussed in Cuoco et al., (2020). This study highlights the presence of an additional inflow in the shallow volcanic aquifer from the Mt. Maggiore carbonate aquifer in correspondence with the Ferrarelle mineral water area.

## Materials and methods

Water samples were collected monthly from October 2017 to January 2019 from eight monitoring or productive wells tapping the FGS for mineral water bottling activity (Fig. 1). D1, D2 and D3 are the deeper wells tapping the carbonate portion of the aquifer, collecting the most mineralized groundwater of the FGS. These wells are only used for CO_2_ collection and the tapped groundwater is not included in the bottling activities.

The monitored wells are the same as the ones reported in Cuoco et al., (2020) and their technical details are reported in Appendix Table 3. Field measurements were performed following the procedures described in Cuoco et al., (2020). Temperature, pH and Electric Conductivity (EC, with automatic compensation to 20 °C) were measured in situ using portable probes (WTW pH/cond 340i). Precision and accuracy were tested against certificated materials; the uncertainties were confirmed in the range of 1%. ORP was measured by means of a Hanna HI991002 meter equipped with an HI1297 probe (Ag/AgCl electrode); the detected values were then converted to Eh by summing + 200 mV to the field measurements (Ryan, 2014), with precision being better than 5%. The content of HCO_3_^−^ was measured in the field by means of 0.1 M HCl titration using a methyl orange indicator. All samples were filtered with 0.45 μm Minisart sterile cellulose acetate membrane filters and separated into three aliquots in polyethylene bottles: one sample was stored directly for major anions analyses, a second was acidified with a few drops of ultrapure HNO_3_ (Merck^®^) for major cations analyses, and a third aliquot was acidified up to 1% nitric acid (Merck^®^) for ICP-MS analyses.

Major elements were analyzed by means of ion chromatography (Dionex DX-120) following EPA methods 300.1 and 300.7. Charge imbalances were less than ± 3%. Precision and accuracy for the IC analyses was tested against the certified standard solution (SANGAMON-03 certified reference material) and was better than 8%. Total Fe, As and the remaining minor and trace elements were analyzed through ICP-MS (Agilent 7500ce) equipped with interference/reaction cell to reduce polyatomic interferences (ORS tech.). A Tuning Solution (AGILENT^®^) was used to check instrument performance and ensure that sensitivity and interference parameters were optimal. Polyatomic interferences on ^75^As were neutralized in the ORS system through Collisional-Induced Dissociation by He gas. ^56^Fe interferences were neutralized in the ORS system through Ion–Molecule Reaction by H_2_ gas. Interference Check Solutions (AGILENT^®^) were used to verify the efficient functioning of the ORS system.

Instrumental drift was monitored in continuum through Y-Tb internal standard with constant concentration. The analytical precision and accuracy for repeated quantifications of sample solution, international and internal standards (Agilent solutions EPA 200.8 Validated Standards) were better than 10%. Detected concentrations exceeded the limits of detection (LOD) and quantification (LOQ) by at least one order of magnitude, following Long and Winefordner (1983).

## Results and discussion

### Geochemistry of sampled groundwater

**Fig. 2:**
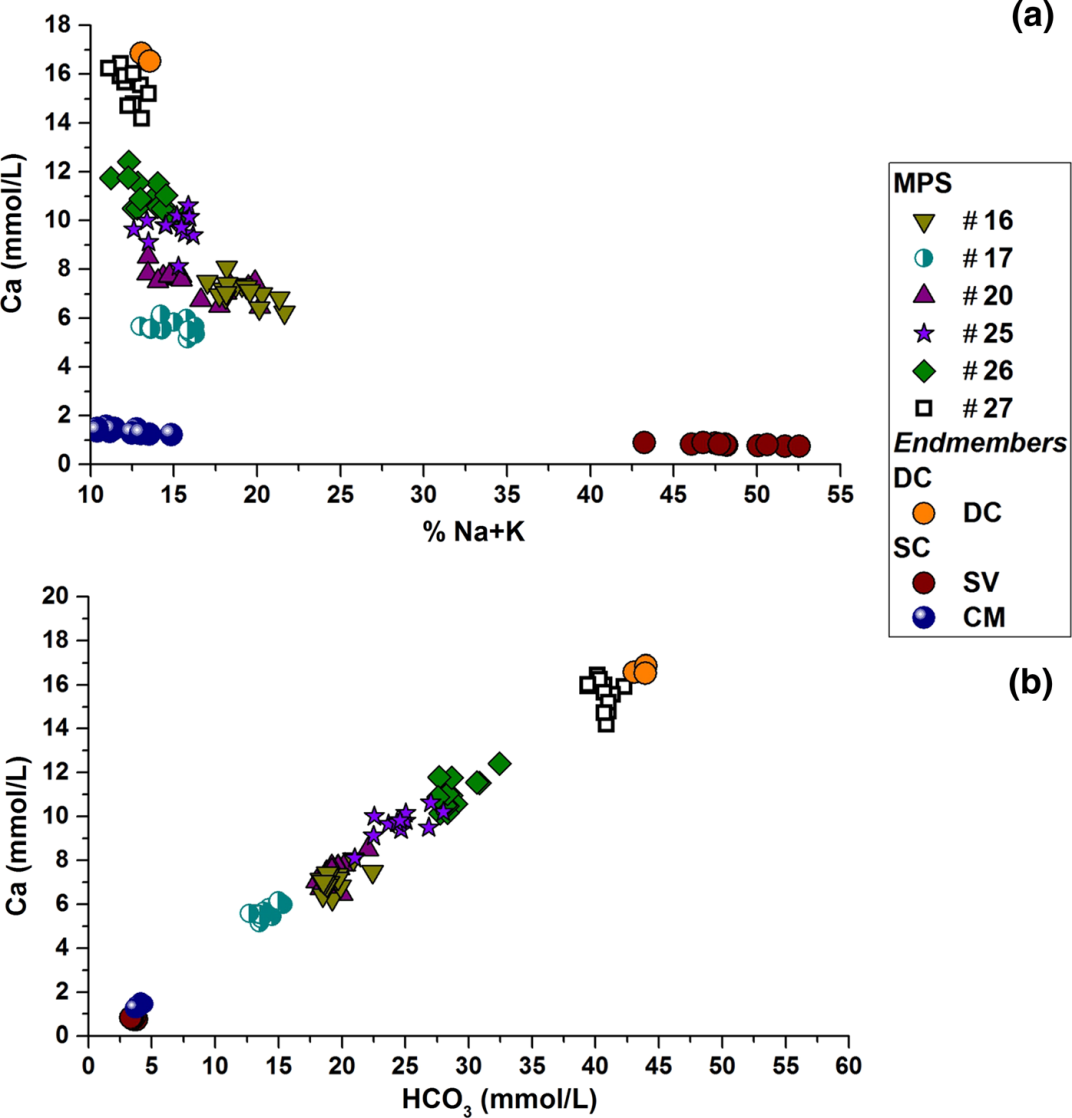
**a** Ca vs. percentage of alkali ions on total major metals. This scatter plot allows the three endmembers in the FGS to be detected. **b** The linear mixing function between deep (DC) and shallow aquifers (SV and CM)

Chemical analyses of sampled waters are summarized in Table 1. Figure 2a plots the molar percentage of alkali ions (% Na + K) against the Ca^2+^ content. This figure allows the different endmembers present in the final collected water to be identified. The deep, highly mineralized (EC ~ 3400 µS/cm) endmember (DC) has the highest amount of dissolved CaCO_3_ in solution (HCO_3_^−^ ~ 98%) due to the high hydrolysis rate of the carbonate host rock triggered by large amounts of CO_2_ (10 mmol/L). DC samples also have the highest Ca^2+^ (17 mmol/L, % Ca ~ 86%) and the lowest % Na + K (14%). Despite having the lowest % Na + K, the DC samples are characterized by the highest concentrations (in absolute scale) of alkali and alkali earth metals Fe, Mn, B and As (Tables 1 and 2). The DC chemical composition is typical of the carbonate aquifer at the basement of FGS (Cuoco et al. 2020). The presence of alkali and alkali earth metals derives from the interaction of water with volcanic rocks overlying or intruded within the carbonate basement improved by the presence of CO_2_–rich waters. Two more endmembers of the mixing were identified in shallow aquifers: (1) groundwater from the carbonate aquifer of Mt. Maggiore (CM) being HCO_3_^−^ - Mg^2+^, Ca^2+^ type (% HCO_3_^−^ ~ 82%, Mg^2+^ + Ca^2+^ ~ 88%) and low mineralized (~ 435 µS/cm); and (2) the volcanic aquifer of Roccamonfina Volcano (SV) being HCO_3_^−^- Na^+^ + K^+^ type (% HCO_3_^−^ ~ 88%, Na^+^ + K^+^ ~ 43–52%) and low mineralized (~ 430 µS/cm). The combination of the shallow volcanic and the carbonate aquifers is defined as a shallow component. The mixing between the deep and shallow components occurs along faults due to the hydraulic connection between shallow low-mineralized aquifers and rising CO_2_-rich waters from the carbonate aquifer (Cuoco et al., 2020; Giordano et al., 1995; Viaroli et al., 2018). The different relative percentages of the endmembers give rise to different chemical compositions of pumped waters. The contribution of the mineralized aquifers increases with depth and/or the closeness to faults.

**Table 1:** Summary of chemical analyses. nd: non detectable

		T (°C)	pH	EC (μS/cm)	Eh (mV)	HCO_3_^−^ (mmol/L)	Cl^−^ (mmol/L)	NO_3_^−^ (mmol/L)	SO_4_^2−^ (mmol/L)	Na^+^ (mmol/L)	K^+^ (mmol/L)	Mg^2+^ (mmol/L)	Ca^2+^ (mmol/L)
W16	Min	14.5	5.90	1678	293	18.3	0.4	0.05	0.04	2.3	1.1	0.9	6.2
	Max	16.4	6.18	1756	382	22.4	0.6	0.1	0.06	2.9	1.4	1.4	8.1
	Mean	15.4	6.03	1716	327	19.5	0.5	0.1	0.05	2.7	1.3	1.2	7.1
	St. dev	0.5	0.07	21	226	1.1	0.0	0.03	0.005	0.2	0.1	0.2	0.5
W17	Min	15.0	5.87	1260	276	13.1	0.5	0.3	0.06	1.4	0.6	0.7	5.2
	Max	16.4	6.12	1452	369	15.4	0.6	0.3	0.07	1.8	0.8	1.0	6.2
	Mean	15.7	6.00	1319	327	14.1	0.5	0.3	0.06	1.6	0.7	0.9	5.7
	St. dev	0.4	0.07	58	224	0.7	0.0	0.03	0.005	0.1	0.1	0.1	0.3
W20	Min	15.7	5.80	1621	301	18.0	0.5	0.1	0.02	1.9	0.8	0.8	6.5
	Max	17.5	6.09	1784	427	22.1	0.7	0.2	0.04	2.8	1.5	1.3	8.5
	Mean	16.4	5.94	1719	342	19.4	0.5	0.1	0.03	2.2	1.1	1.1	7.4
	St. dev	0.5	0.09	52	232	1.0	0.1	0.02	0.004	0.3	0.2	0.2	0.6
W25	Min	15.8	6.01	1795	233	21.0	0.4	nd	0.02	1.8	1.3	0.9	8.1
	Max	18.6	6.22	2290	290	28.0	0.6	nd	0.04	2.8	1.8	1.6	10.6
	Mean	17.2	6.12	2115	265	24.8	0.5		0.03	2.4	1.5	1.4	9.6
	St. dev	1.0	0.09	150	218	2.1	0.04		0.005	0.4	0.2	0.2	0.7
W26	Min	15.0	6.03	2250	270	27.8	0.5	0.01	0.02	2.2	1.1	1.5	10.1
	Max	16.5	6.29	2500	326	32.5	0.6	0.06	0.03	2.9	1.7	2.4	12.4
	Mean	15.8	6.17	2386	304	29.1	0.5	0.03	0.03	2.6	1.5	1.9	10.9
	St. dev	0.5	0.09	75	218	1.4	0.0	0.01	0.004	0.2	0.2	0.2	0.7
W27	Min	16.0	6.26	2860	170	34.9	0.5	nd	0.01	3.1	1.4	2.0	13.3
	Max	17.4	6.42	3270	257	42.3	0.7	nd	0.02	3.7	1.8	3.2	16.4
	Mean	16.5	6.33	3178	219	40.5	0.6		0.02	3.4	1.7	2.6	15.3
	St. dev	0.5	0.05	125	220	1.8	0.0		0.002	0.2	0.1	0.3	0.9
DC	D1	16.5	6.2	3305.00	163	43.1	0.7		0.02	3.6	2.2	2.1	16.6
	D2	17.3	6.2	3329.00	170	44.0	0.7		0.01	3.6	2.2	2.4	16.9
	D3	16.6	6.2	3490.00	161	43.9	0.7		0.01	3.7	2.2	2.2	16.5
SV	Min	14.2	6.31	415	284	3.3	0.4	0.07	0.03	1.2	0.6	0.2	0.8
	Max	16.3	6.57	449	343	3.8	0.5	0.12	0.05	1.5	0.8	0.3	0.9
	Mean	15.0	6.40	432	310	3.6	0.4	0.11	0.04	1.4	0.7	0.3	0.8
	St. dev	0.7	0.07	13	221	0.2	0.0	0.02	0.005	0.1	0.1	0.0	0.1
CM	Min	13.8	7.43	421	232	3.8	0.5	0.004	0.09	0.4	0.1	0.8	1.2
	Max	17.6	7.77	459	319	4.4	0.5	0.02	0.12	0.6	0.1	0.9	1.6
	Mean	15.4	7.66	439	276	4.0	0.5	0.01	0.10	0.5	0.1	0.9	1.4
	St. dev	1.4	0.13	13	225	0.2	0.0	0.01	0.01	0.1	0.0	0.1	0.1

**Table 2:** Summary of chemical analyses (trace elements). nd: non detectable

		Li (µmol/L)	B (µmol/L)	Fe (µmol/L)	As (µmol/L)	Rb (µmol/L)	Sr (µmol/L)	Cs (µmol/L)	Ba (µmol/L)
W16	Min	10.8	21.8	1.1	0.13	1.90	6.68	0.03	0.16
	Max	12.5	25.6	1.9	0.15	2.26	7.66	0.04	0.41
	Mean	11.7	23.2	1.6	0.14	2.07	7.17	0.03	0.20
	St. dev	0.6	1.2	0.2	0.005	0.12	0.33	0.003	0.07
W17	Min	8.3	19.2	0.01	0.08	1.65	6.73	0.08	0.25
	Max	9.2	29.3	0.2	0.09	2.34	7.83	0.15	0.46
	Mean	8.7	22.7	0.1	0.08	1.98	7.24	0.12	0.32
	St. dev	0.3	3.0	0.04	0.005	0.20	0.38	0.03	0.06
W20	Min	7.7	35.5	0.04	0.13	1.42	6.67	0.04	0.32
	Max	9.7	45.7	0.2	0.15	1.75	8.00	0.05	0.38
	Mean	8.7	40.2	0.1	0.14	1.58	7.29	0.05	0.35
	St. dev	0.7	4.1	0.03	0.01	0.11	0.42	0.004	0.02
W25	Min	10.4	38.5	23.9	0.11	1.14	7.69	0.36	0.88
	Max	15.2	59.1	50.1	0.22	1.59	11.52	0.52	1.57
	Mean	13.7	53.9	39.6	0.17	1.37	10.37	0.46	1.25
	St. dev	1.5	6.0	8.4	0.04	0.13	1.17	0.05	0.21
W26	Min	14.5	57.5	3.7	0.06	1.14	9.89	0.33	0.88
	Max	17.4	70.7	10.8	0.11	1.47	10.97	0.39	1.03
	Mean	15.6	61.5	6.9	0.08	1.28	10.45	0.36	0.95
	St. dev	0.8	3.7	2.6	0.02	0.08	0.32	0.02	0.05
W27	Min	15.6	55.8	20.1	0.06	2.01	15.69	0.01	1.40
	Max	23.1	76.4	99.0	0.15	2.57	17.71	0.08	1.87
	Mean	20.0	66.6	73.8	0.14	2.17	16.36	0.06	1.74
	St. dev	2.5	6.5	21.9	0.03	0.15	0.63	0.02	0.12
DC	D1	12.7	74.0	80.4	0.5	1.8	17.2	0.4	2.3
	D2	21.0	97.1	144.4	0.3	2.1	21.1	0.9	2.4
	D3	26.7	125.5	130.3	0.6	2.0	20.2	0.9	2.2
SV	Min	4.1	7.9	0.01	0.11	0.72	1.16	0.06	0.09
	Max	4.6	8.8	0.06	0.12	0.93	1.31	0.07	0.12
	Mean	4.4	8.4	0.04	0.12	0.84	1.27	0.07	0.10
	St. dev	0.1	0.3	0.02	0.002	0.07	0.04	0.01	0.01
CM	Min	0.5	1.0	0.02	0.02	0.18	1.19	0.004	0.06
	Max	0.8	1.7	0.12	0.02	0.23	1.35	0.01	0.09
	Mean	0.6	1.3	0.07	0.02	0.20	1.27	0.01	0.07
	St. dev	0.2	0.2	0.03	-	0.01	0.06	0.003	0.01

The linear mixing function reported in Fig. 2b (*R* = 0.99 *p* < 0.001) reproduces the FGS hydrogeochemistry between the deep mineralized aquifer (DC) and the shallow low mineralized aquifers (SV and CM). Even though Ca^2+^ and HCO_3_^−^ are not two conservative elements in this system, the strong linear function points out the dilution effect of the DC in the mixing with shallow and low mineralized SV and CM groundwater. For the records, any quantitative computation of component percentages is avoided. The last two components in Fig. 2 can be combined into a single endmember, confirming hydrodynamics related to hydraulic contact between deep and shallow aquifers in the FGS, as discussed in Cuoco et al., (2020) and Viaroli et al., (2018).

### Fe and As in solution throughout the mixing

**Fig. 3:**
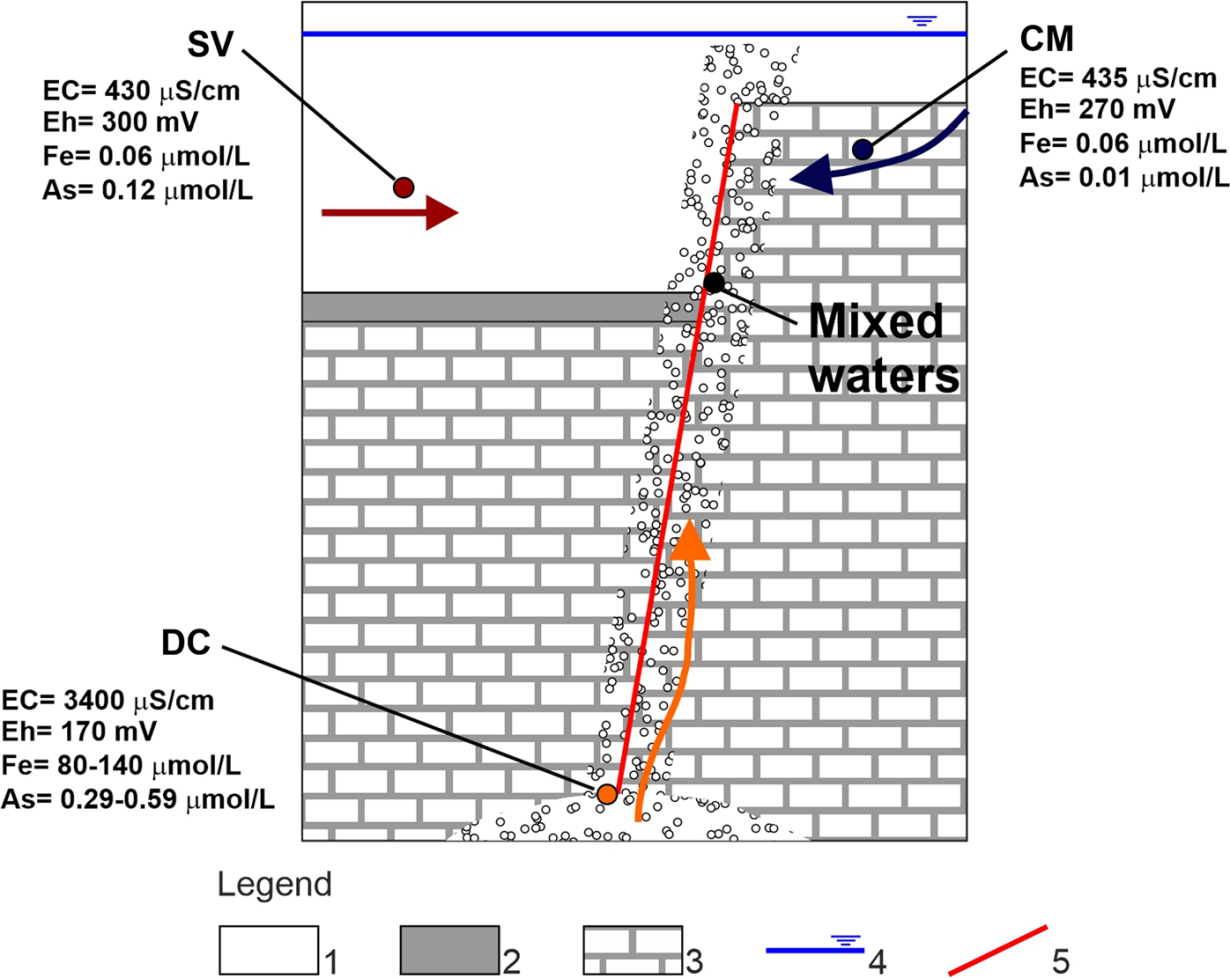
Conceptual scheme of endmembers and mixing dynamic occurring in the Ferrarelle Groundwater System. (SV) shallow volcanic aquifer, (CM) Mt. Maggiore carbonate aquifer, (DC) mineralized aquifer. Legend: (1) Volcanic deposits, (2) Clay deposits, (3) Carbonate basement, (4) Groundwater level, (5) Fault

The groundwater mixing can generate the combined effect of dilution and redox re-equilibration; the hydrodynamics which can allow this process is schematized in Fig. 3. The Eh detected in the DC samples was ~ 170 mV, whereas it was higher (~ 300 mV) in SV and CM. This finding matches a significant difference in Fe and As concentrations (Fig. 3). In the three deep wells (D1, D2, and D3), different Fe concentrations were detected (Table 1): D1 = 80.4 µmol/L; D2 = 144 µmol/L and D3 = 130 µmol/L. Concentrations were found to be lowest in D1 not only of Fe but also of Cs (0.43 µmol/L in D1 vs. ~ 0.90 µmol/L similar in wells D2 and D3), B (74.1 µmol/L in D1 vs. 97.1–125.5 µmol/L in D2 and D3) and Li (13 µmol/L in D1 vs. 21–26 µmol/L in D2 and D3). These differences in trace elements concentrations may be related to local geological settings (e.g., presence of intruded volcanic rocks in the carbonate basement) and hydrochemical dynamics within the carbonate aquifer.

The Fe content is significantly lower in the shallow aquifers (< 0.1 µmol/L) compared to the deep aquifer (ranging between 80 and 144 µmol/L). The geochemical evolution of DC along its flow path implies oxygen consumption in the redox reactions and, as a consequence, the leached iron is present in the reduced form Fe(II), which, being soluble, can be enriched in groundwater solution (Appelo & Postma, 1993). The strong volcanites leaching in DC also produces the highest As concentrations detected (0.29–0.59 µmol/L). On the contrary, the low alteration rate in the shallow aquifers results in lower As concentration both in SV (As = 0.12 µmol/L) and CM (As = 0.06 µmol/L) (Fig. 3).

The hydraulic connection between DC and the shallow aquifers produces important changes in redox equilibria as observed in the mixed groundwater samples. The Fe concentration linearly decreases with EC (Pearson's R = 0.82, *p* < 0.001) from 100 µmol/L up to 0.01 µmol/L.

The inverse correlation between Ca^2+^ and Eh results in a Pearson's *R* = − 0.80 (*p* < 0.001), because the more Ca^2+^ rich groundwater (DC) has the lowest Eh values; with the increase in the dilution effect due to the mixing with shallow less mineralized aquifers in oxidant conditions, the Ca^2+^ concentration decreases and the Eh increases. The same process affects the Fe concentrations, as confirmed by the inverse correlation between Fe and Eh (*R* = − 0.85, *p* < 0.001), confirming that the redox conditions change due to the dilution effect.

**Fig. 4:**
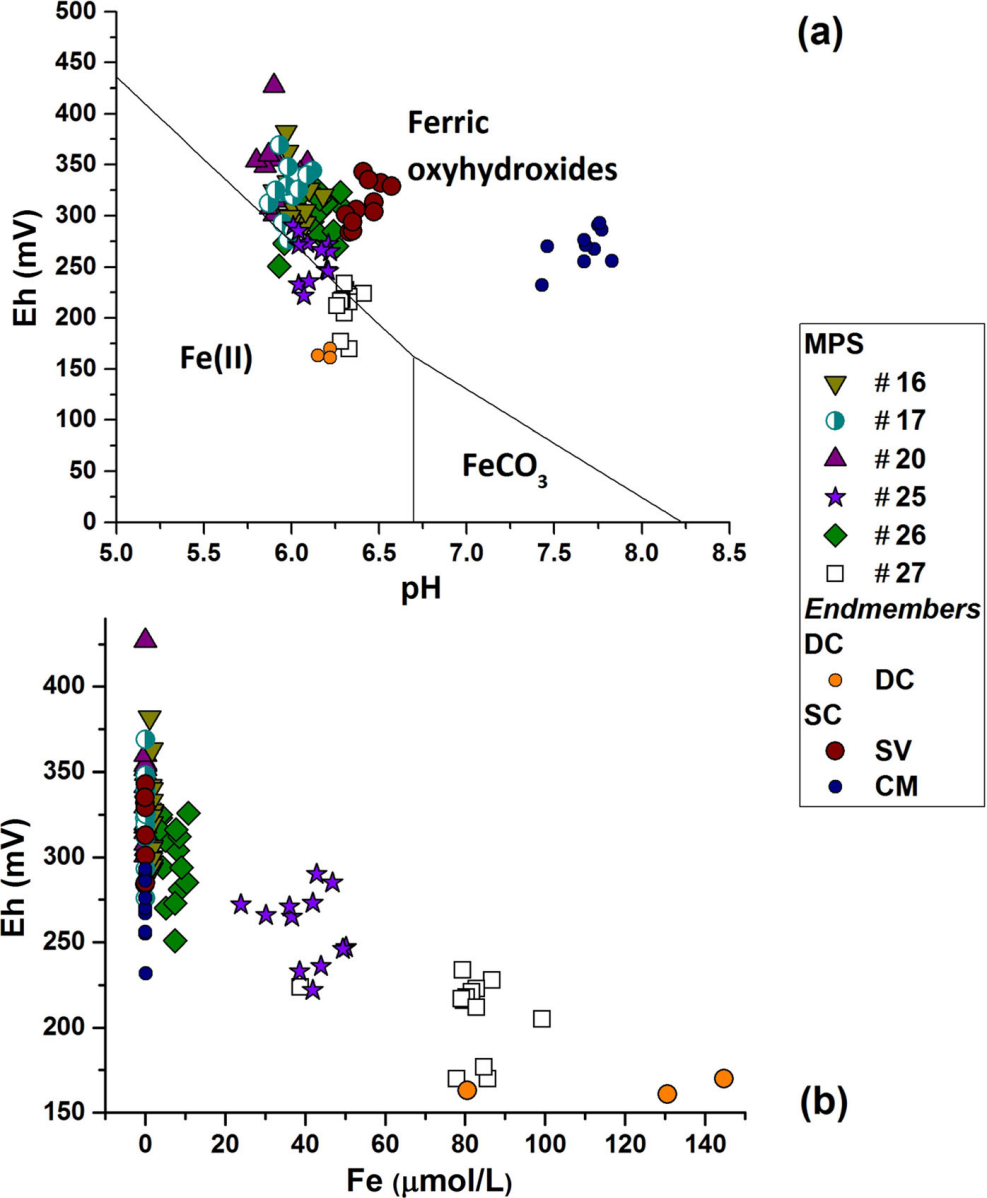
**a** pH-Eh diagram for Fe–H_2_O–O_2_–CO_2_ system (Whittemore & Langmuir, 1975) reported in the range of detected pH and Eh values. The Fe(II)/Fe(III) boundary for analyzed samples is visible in the plot. **b** in agreement with (**a**) the samples with the relevant Fe concentrations have soluble Fe(II) as the predominant species

The Fe speciation is strictly related to the Eh variations. According to the PHREEQC computer code, Fe(II) can be detected as appreciable iron species in W25, W27, W26 samples in which the higher DC components are identified. The pH-Eh diagram for the Fe–H_2_O–O_2_–CO_2_ system (Fig. 4a, Whittemore & Langmuir, 1975) confirms this elaboration; in fact, these samples lie close to the Fe(II) and Fe(III) equilibrium boundary line. In Fig. 4b, Eh is plotted against total Fe; the higher Fe concentrations are related to the lower Eh values, confirming that the redox equilibrium agrees with the iron chemical species as soluble Fe(II).

**Fig. 5:**
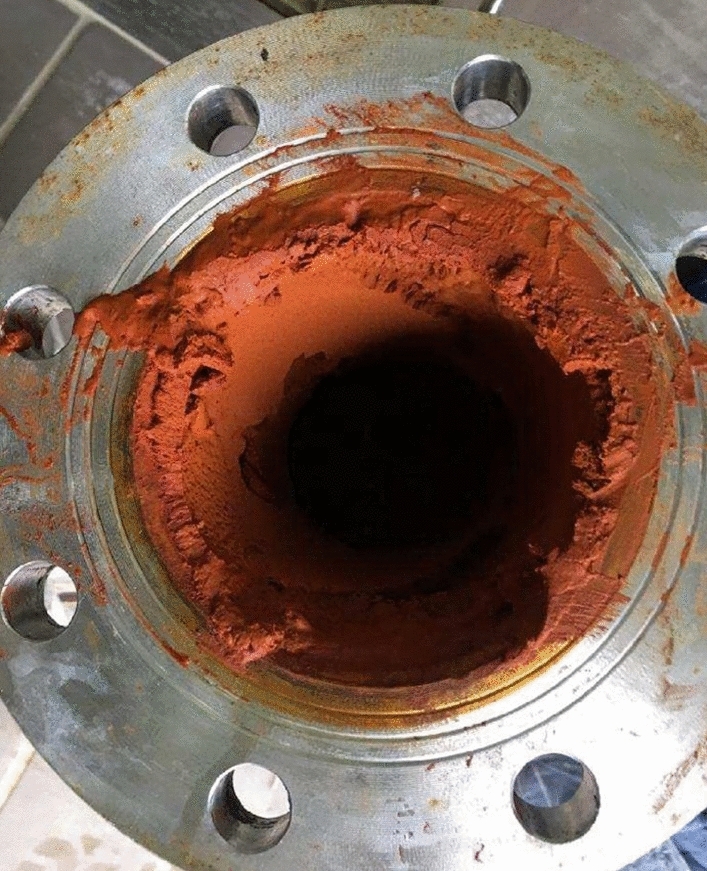
Fe oxohydroxides precipitated inside a drop pipe section of a mineral water well

The iron oxidation is due to the oxidizing shallow components which play a key role as electrons acceptors in this redox equilibrium (e.g., Appelo & Postma, 1999; Grenthe et al., 1992). The oxidation of Fe(II) to Fe(III) produces insoluble ferric oxohydroxides formation (Stumm & Morgan, 1996) (Fig. 5). At the same time, As adsorption on ferric oxohydroxides induces a co-precipitation, producing both As and Fe removal from groundwater (Dzombak & Morel, 1990; Pierce & Moore, 1982). As concentrations vary from 0.22 µmol/L in the more saline and reducing waters up to 0.1–0.2 µmol/L in the lower mineralized mixed groundwater samples characterized by oxidant conditions.

**Fig. 6:**
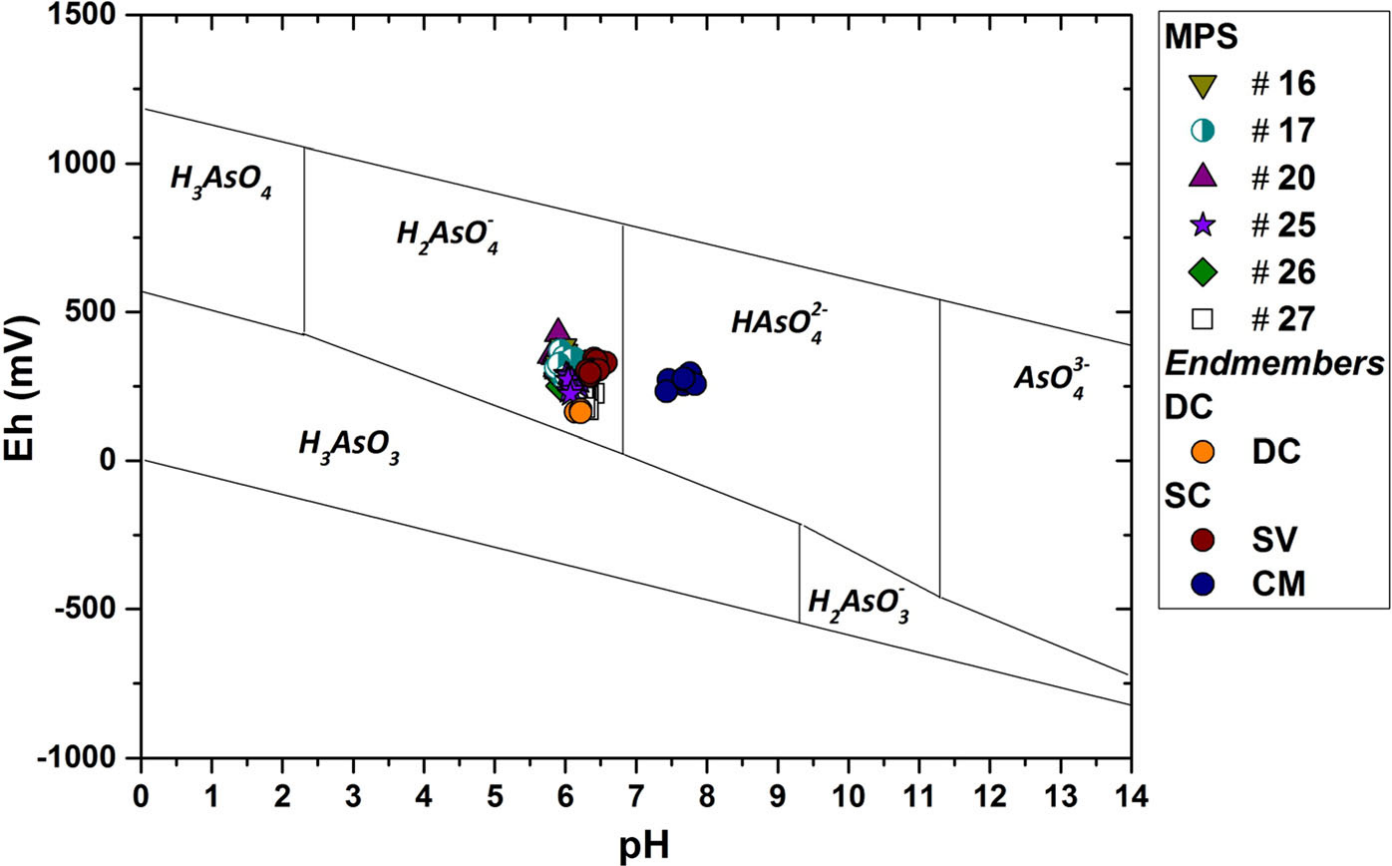
pH-Eh diagram for As species. The oxidation state of arsenic is As(V), which is mainly present as H_2_AsO_4_^−^ ion.

The predominant As chemical species in solution was estimated through thermodynamic data proposed by Nordstrom and Archer (2003) and the results are shown in the Eh–pH diagram (Fig. 6). Data suggest that arsenic is mostly present in the oxidized form as H_2_AsO_4_^−^.

Conversely, the predominant As species in CM is HAsO_4_^2−^, according to the more alkaline groundwater of the Mt. Maggiore carbonate aquifer (Table 1) and higher dissociation value (pKa_2_ = 6.96). The presence of HAsO_4_^2−^in the studied system is negligible due to the very low As concentration in CM (Table 1).

Once the hydrodynamics and the chemical form of As and Fe are defined, it is possible to define the chemical reactions pattern which describes the self-depuration dynamics.

### Chemical reactions pattern, key parameters and mixing functions.

The oxygen in solution leads the oxidation process of Fe(II). The dissolved oxygen is given by the Henry constant

1 $$K_{{{\text{H}}\left( {{\text{O}}_{2} } \right)}} = \frac{{\left[ {{\text{O}}_{2} } \right]}}{{P_{{O_{2} }} }}$$

A first approximation is made by considering the activity and fugacity coefficients ~ 1, thus the activity of solutes can be approximated to their molar concentration and the fugacity of oxygen to its partial pressure $P_{{O_{2} }}$. When DC mixes with SV and CM groundwater, the oxygen in solution increases and reacts as follows

2 $$\begin{gathered} 4{\text{Fe}}^{2 + } + {\text{O}}_{2} + 10{\text{H}}_{2} {\text{O}} \leftrightarrow 4{\text{Fe}}({\text{OH}})_{3} + 8{\text{H}}^{ + } \hfill \\ \left( {\Delta G = - 115.51\frac{{{\text{KJ}}}}{{{\text{mol}}}}; {\text{Log}}K_{eq} = 10^{ - 2} } \right) \hfill \\ \end{gathered}$$

The kinetics of this reaction, for the pH values comparable with those of sampled waters (6.00–7.80), depend on a rate law of first order with respect to the concentrations of both Fe(II) and O_2_. The oxidation halftime was estimated to be less than 30 min (Geroni & Sapsford, 2011; Sung & Morgan, 1980). The speciation of Fe(II) in natural waters is still a matter of debate and suffers from several uncertainties. The main Fe species in samples with the lowest values of pH and Eh was computed by PHREEQC as Fe^2+^ and FeHCO_3_^+^ complex (over 99%)**.** The R-Pearson between FeHCO_3_^+^ and Eh was − 0.86, thus the oxidation of Fe(II) seems reasonable; however King (1998) observed an oxidation kinetics very slow for this compound to the point where he neglected its oxidation reaction, and in addition the dissociation of this complex is not a spontaneous reaction. Several authors assert the non-effective existence of the FeHCO_3_^+^ complex (Bruno et al., 1992; Singer & Stumm, 1970). Lemire et al., (2013) affirmed that at least the complex is very weak and its potential field of stability is very narrow to the one of FeCO_3_ (siderite). Fe^2+^ from siderite in the presence of oxygen is oxidized as Fe(III) oxohydroxides (Renard et al., 2017), thus we consider the reaction (2) valid to describe the whole process of Fe(II) redox transformation to Fe(III). In light of the lack of defined knowledge of the distribution of Fe(II) complexes in natural waters, and considering the observation made in the present study on Fe speciation through Fig. 4, we are inclined to agree with the conclusion suggested by Singer and Stumm (1970) stating that Fe^2+^ is the only significant Fe species in carbonate-bearing waters, thus we approximate [Fe]$\cong$[Fe^2+^].

Once iron oxohydroxides are produced by reaction (2), the arsenate ions are adsorbed as follows

3 $$\begin{gathered} {\text{Fe}}({\text{OH}})_{3} + {\text{H}}_{2} {\text{AsO}}_{4}^{ - } + {\text{H}}^{ + } \to ~{\text{Fe}}({\text{OH}})_{2} - {\text{H}}_{2} {\text{AsO}}_{4} + ~{\text{H}}_{2} {\text{O}} \hfill \\ ({\text{Log X}}_{{{\text{ads}}}} = 4.50) \hfill \\ \end{gathered}$$

where ${\mathrm{\rm X}}_{ads}$ is the surface complexation constant given by Elyahyaoui et al. (2016) and by Goldberg (2011). Raven et al. (1998) observed that the absorption of arsenate is fairly fast and is complete four hours after reaction initiation. Fuller et al. (1993) indicated the very rapid adsorption of arsenate in the first five minutes of the reaction.

Combining the equilibrium constants of reactions (2) and (3), approximating $\left[ {{\text{H}}_{2} {\text{AsO}}_{4}^{ - } } \right]\left[ {{\text{As}}} \right]$, $\left[ {{\text{Fe}}^{2 + } } \right]\left[ {{\text{Fe}}} \right]$, we obtain the following expression

4 $$\left[ {As} \right] = \left( {\frac{{K_{eq} \left[ {O_{2} } \right]}}{{\left[ {H^{ + } } \right]^{8} {\text{\rm X}}^{1/4} }}} \right)^{1/4} \left[ {Fe} \right]$$

**Fig. 7:**
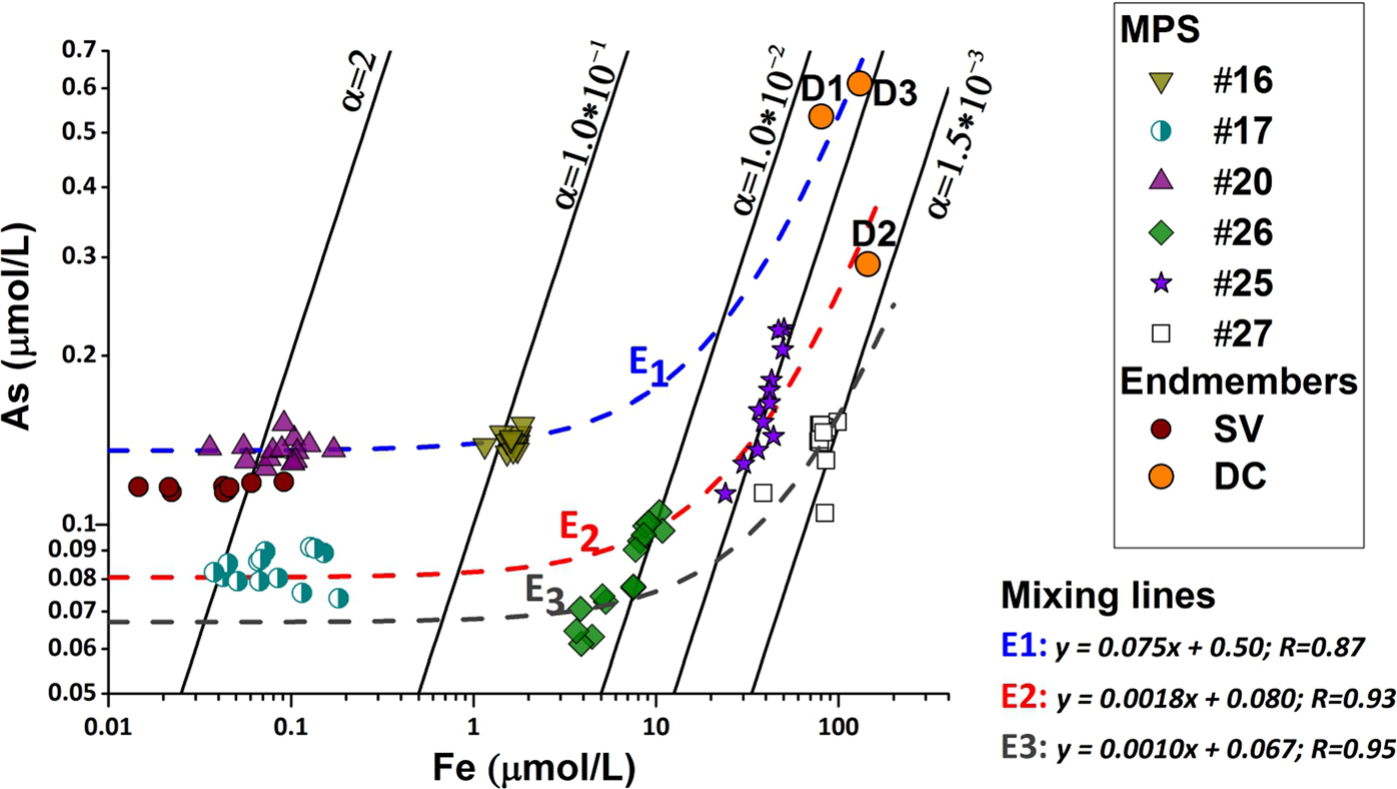
Detected Fe and As concentration fit the elaborated theoretical functions.

We identify $\alpha = \left( {\frac{{K_{{{\text{eq}}}} \left[ {O_{2} } \right]}}{{\left[ {H^{ + } } \right]^{8} {\text{\rm X}}^{1/4} }}} \right)^{1/4} ,$ in As vs. Fe binary plot. It represents the angular coefficient of the line describing the oxidation-adsorption equilibrium. In Fig. 7, both [As] versus [Fe] and the lines following the relation (4) at different $\alpha$ are plotted. The more mineralized samples (W25, W26 and W27) fit with theoretical lines (*χ*^2^ test for *p* < 0.01). This result was expected, because for $\alpha <$ 0.02, samples have redox conditions that allow Fe (II) in solution, in agreement with the results shown in Fig. 4. Since Fe (II) is in solution, Fe(II) oxidation triggers the formation of insoluble oxohydroxides which cause As adsorption and co-precipitation from the water solution. In light of these results, the possible presence of competing ions for sorption sites on iron precipitates does not seem to generate a significant deviation of analytical data from the proposed theoretical reactions pattern.

With the increase in $\alpha$, the removal of Fe and As improves; this equilibrium is defined by the ratio of dissolved oxygen and pH. The mixing with shallow waters with highest pH and oxygen content activates Fe and As co-precipitation simultaneously. When the amount of Fe(II) in solution becomes negligible, the As concentration becomes independent of the total Fe amount (W16, W17, W20). In light of the defined reaction pattern, the deep vs. shallow aquifers mixing functions can be obtained. In Fig. 7, three different mixing lines E1, E2, and E3 were computed by sample alignments on the plot. The fit is significant (*R* > 0.87, *p* < 0.001). The different intercept values and angular coefficients should be due to inhomogeneous redox equilibria in the FGS, similarly to the different As/Fe ratio in the D2 well compared to the D1 and D3 wells.

The W26 samples (Fig. 7) fit with both E2 and E3 lines. The scattered results are influenced by the pumping rate of the well (Ferrarelle Data Report, personal communication) during the monitoring period. Increasing the pumping rate of the well induces the upraise of the deeper mineralized and reductive groundwater, varying the mixing equilibria. This produces a shift in Eh value, Fe and As content according to the above described chemical dynamics.

The fitting of samples on the E lines, observed through the evolution of oxidation-co-precipitation dynamics (i.e., the increase in α parameters) reproduces the evolution of groundwater chemical equilibriums in the described mixing dynamics.

## Conclusion

High concentrations of Fe and As are present in the Ferrarelle Groundwater System deep aquifer, in agreement with reducing conditions of groundwater. The vertical mixing, between shallow groundwater in an oxidizing condition and deep (CO_2_-rich) waters in reducing conditions produces Fe(II) oxidation, forming iron oxohydroxides. These solids have the capability to adsorb As and then precipitate, removing both Fe and As from solution. This is a self-depuration process produced by the sequence of oxidation—adsorption—coprecipitation which represent the natural dynamics of decontamination of the deep volcanic aquifers in central-southern Italy and also in similar hydrogeological frameworks worldwide. Reactions governing this series of geochemical processes are described in the present investigation. Approximating the water solution to an ideal behavior did not influence the good fit of data in the frame of the theoretical reactions pattern proposed. The process of Fe-As self-removal is governed by the ratio of dissolved oxygen and pH; the higher this ratio becomes, the more Fe-As coprecipitation there is. Over the Fe(II)/Fe(III) boundary, the total Fe is removed from solution due to its precipitation, and the potential capability of water to remove As from solution decreases. Starting from this point, As concentration becomes independent of Fe concentration.

In the Ferrarelle Groundwater System, Fe and As concentrations are therefore naturally reduced. It proves to be a good natural test laboratory for hydrogeochemical processes governing the mixing dynamics among highly mineralized and CO_2_ saturated groundwater and shallow freshwaters. The mixing lines can be reproduced in an Fe vs. As plot, considering the relationship between the chemical parameters and the equilibrium constants of the reaction related to Fe oxidation and As adsorption. The co-presence of reduced Fe in solution and As, and very likely, other absorbable elements, is a potential advantage for water self-depuration by oxygenation due to exposition to atmosphere and/or mixing with more oxidizing water bodies. These dynamics reveal that this single process can efficiently remove both Fe(II) and As from solution. The outcomes discussed in this paper can be a useful reference in the identification, exploitation, and management of natural mineral water for bottling activities. They will undoubtedly be helpful in the definition of the occurrence of self-removal geochemical processes and in the understanding of the key parameters to observe in order to tap the groundwater with the minimal and/or effective treatment requirement.

## Appendix

See Table 3

**Table 3:** Technical specifications for drilling and constructing the sampled water wells

ID	Elevation (m a.s.l.)	Depth (m)	Elevation of the well bottom (m a.s.l.)	Screen interval (m a.s.l.)	Tapped aquifer
W26	146	146	0	From 19 to 0	Carbonate
W20	97	18	79	From 86 to 79	Volcanic
W16	96.5	17.5	79	From 86 to 79	Volcanic
W17	100.5	33	67.5	From 85.5 to 67.5	Volcanic
W25	104	129	− 16	From 34 to − 16	Volcanic and carbonate
W27	103	138	− 35	From 43 to − 35	Volcanic
D1	139.8	295	− 155.2	From − 126 to − 155.2	Carbonate
D2	105.9	120	− 14.1	From 14 to − 14.1	Carbonate
D3	90	266.5	− 176.5	From − 101 to − 176.5	Carbonate
SV	122.4	63	59.4	From 100 to − 59.4	Volcanic
CM	144	46	98	From 109 to 98	Carbonate
