# Correction to: Fe and As geochemical self-removal dynamics in mineral waters: evidence from the Ferrarelle groundwater system (Riardo Plain, Southern Italy)

**DOI:** 10.1007/s10653-021-00989-w

**Published:** 2021-06-26

**Authors:** Emilio Cuoco, Stefano Viaroli, Vittorio Paolucci, Roberto Mazza, Dario Tedesco

**Affiliations:** 1 Istituto Nazionale Di Geofisica E Vulcanologia, Osservatorio Vesuviano, Via Diocleziano 328, 80124 Napoli, Italy; 2 Dipartimento Di Scienze, Università degli Studi Roma Tre, Largo S. Leonardo Murialdo 1, 00146 Roma, Italy; 3 Ferrarelle S.p.A., Contrada Ferrarelle, Riardo, Italy; 4 Dipartimento Di Scienze E Tecnologie Ambientali, Biologiche E Farmaceutiche, Università Della Campania “L. Vanvitelli”, Via Vivaldi 43, 81100 Caserta, Italy

## Correction to: Environ Geochem Health 10.1007/s10653-021-00891-5

In the original publication of the article, the "Ca2 + (Calcium) column" was incorrectly included in Table 2 with the trace elements. However, it should be in Table 1 with the other major ions.

The correct Tables 1 and 2 are given below.

**Table 1:** Summary of chemical analyses

		T	pH	EC	Eh	HCO_3_^−^	Cl^−^	NO_3_^−^	SO_4_^2−^	Na^+^	K^+^	Mg^2+^	Ca^2+^
		°C		µS/cm	mV	mmol/L	mmol/L	mmol/L	mmol/L	mmol/L	mmol/L	mmol/L	mmol/L
W16	Min	14.5	5.90	1678	293	18.3	0.4	0.05	0.04	2.3	1.1	0.9	6.2
	Max	16.4	6.18	1756	382	22.4	0.6	0.1	0.06	2.9	1.4	1.4	8.1
	Mean	15.4	6.03	1716	327	19.5	0.5	0.1	0.05	2.7	1.3	1.2	7.1
	St. Dev.	0.5	0.07	21	226	1.1	0.0	0.03	0.005	0.2	0.1	0.2	0.5
W17	Min	15.0	5.87	1260	276	13.1	0.5	0.3	0.06	1.4	0.6	0.7	5.2
	Max	16.4	6.12	1452	369	15.4	0.6	0.3	0.07	1.8	0.8	1.0	6.2
	Mean	15.7	6.00	1319	327	14.1	0.5	0.3	0.06	1.6	0.7	0.9	5.7
	St. Dev.	0.4	0.07	58	224	0.7	0.0	0.03	0.005	0.1	0.1	0.1	0.3
W20	Min	15.7	5.80	1621	301	18.0	0.5	0.1	0.02	1.9	0.8	0.8	6.5
	Max	17.5	6.09	1784	427	22.1	0.7	0.2	0.04	2.8	1.5	1.3	8.5
	Mean	16.4	5.94	1719	342	19.4	0.5	0.1	0.03	2.2	1.1	1.1	7.4
	St. Dev.	0.5	0.09	52	232	1.0	0.1	0.02	0.004	0.3	0.2	0.2	0.6
W25	Min	15.8	6.01	1795	233	21.0	0.4	nd	0.02	1.8	1.3	0.9	8.1
	Max	18.6	6.22	2290	290	28.0	0.6	nd	0.04	2.8	1.8	1.6	10.6
	Mean	17.2	6.12	2115	265	24.8	0.5	–	0.03	2.4	1.5	1.4	9.6
	St. Dev.	1.0	0.09	150	218	2.1	0.04	–	0.005	0.4	0.2	0.2	0.7
W26	Min	15.0	6.03	2250	270	27.8	0.5	0.01	0.02	2.2	1.1	1.5	10.1
	Max	16.5	6.29	2500	326	32.5	0.6	0.06	0.03	2.9	1.7	2.4	12.4
	Mean	15.8	6.17	2386	304	29.1	0.5	0.03	0.03	2.6	1.5	1.9	10.9
	St. Dev.	0.5	0.09	75	218	1.4	0.0	0.01	0.004	0.2	0.2	0.2	0.7
W27	Min	16.0	6.26	2860	170	34.9	0.5	nd	0.01	3.1	1.4	2.0	13.3
	Max	17.4	6.42	3270	257	42.3	0.7	nd	0.02	3.7	1.8	3.2	16.4
	Mean	16.5	6.33	3178	219	40.5	0.6	–	0.02	3.4	1.7	2.6	15.3
	St. Dev.	0.5	0.05	125	220	1.8	0.0	–	0.002	0.2	0.1	0.3	0.9
DC	D1	16.5	6.2	3305.00	163	43.1	0.7	–	0.02	3.6	2.2	2.1	16.6
	D2	17.3	6.2	3329.00	170	44.0	0.7	–	0.01	3.6	2.2	2.4	16.9
	D3	16.6	6.2	3490.00	161	43.9	0.7	–	0.01	3.7	2.2	2.2	16.5
SV	Min	14.2	6.31	415	284	3.3	0.4	0.07	0.03	1.2	0.6	0.2	0.8
	Max	16.3	6.57	449	343	3.8	0.5	0.12	0.05	1.5	0.8	0.3	0.9
	Mean	15.0	6.40	432	310	3.6	0.4	0.11	0.04	1.4	0.7	0.3	0.8
	St. Dev.	0.7	0.07	13	221	0.2	0.0	0.02	0.005	0.1	0.1	0.0	0.1
CM	Min	13.8	7.43	421	232	3.8	0.5	0.004	0.09	0.4	0.1	0.8	1.2
	Max	17.6	7.77	459	319	4.4	0.5	0.02	0.12	0.6	0.1	0.9	1.6
	Mean	15.4	7.66	439	276	4.0	0.5	0.01	0.10	0.5	0.1	0.9	1.4
	St. Dev.	1.4	0.13	13	225	0.2	0.0	0.01	0.01	0.1	0.0	0.1	0.1

nd: non detectable

**Table 2:** Summary of chemical analyses (trace elements)

		Li	B	Fe	As	Rb	Sr	Cs	Ba
		µmol/L	µmol/L	µmol/L	µmol/L	µmol/L	µmol/L	µmol/L	µmol/L
W16	Min	10.8	21.8	1.1	0.13	1.90	6.68	0.03	0.16
	Max	12.5	25.6	1.9	0.15	2.26	7.66	0.04	0.41
	Mean	11.7	23.2	1.6	0.14	2.07	7.17	0.03	0.20
	St. dev.	0.6	1.2	0.2	0.005	0.12	0.33	0.003	0.07
W17	Min	8.3	19.2	0.01	0.08	1.65	6.73	0.08	0.25
	Max	9.2	29.3	0.2	0.09	2.34	7.83	0.15	0.46
	Mean	8.7	22.7	0.1	0.08	1.98	7.24	0.12	0.32
	St. Dev.	0.3	3.0	0.04	0.005	0.20	0.38	0.03	0.06
W20	Min	7.7	35.5	0.04	0.13	1.42	6.67	0.04	0.32
	Max	9.7	45.7	0.2	0.15	1.75	8.00	0.05	0.38
	Mean	8.7	40.2	0.1	0.14	1.58	7.29	0.05	0.35
	St. Dev.	0.7	4.1	0.03	0.01	0.11	0.42	0.004	0.02
W25	Min	10.4	38.5	23.9	0.11	1.14	7.69	0.36	0.88
	Max	15.2	59.1	50.1	0.22	1.59	11.52	0.52	1.57
	Mean	13.7	53.9	39.6	0.17	1.37	10.37	0.46	1.25
	St. Dev.	1.5	6.0	8.4	0.04	0.13	1.17	0.05	0.21
W26	Min	14.5	57.5	3.7	0.06	1.14	9.89	0.33	0.88
	Max	17.4	70.7	10.8	0.11	1.47	10.97	0.39	1.03
	Mean	15.6	61.5	6.9	0.08	1.28	10.45	0.36	0.95
	St. Dev.	0.8	3.7	2.6	0.02	0.08	0.32	0.02	0.05
W27	Min	15.6	55.8	20.1	0.06	2.01	15.69	0.01	1.40
	Max	23.1	76.4	99.0	0.15	2.57	17.71	0.08	1.87
	Mean	20.0	66.6	73.8	0.14	2.17	16.36	0.06	1.74
	St. Dev.	2.5	6.5	21.9	0.03	0.15	0.63	0.02	0.12
DC	D1	12.7	74.0	80.4	0.5	1.8	17.2	0.4	2.3
	D2	21.0	97.1	144.4	0.3	2.1	21.1	0.9	2.4
	D3	26.7	125.5	130.3	0.6	2.0	20.2	0.9	2.2
SV	Min	4.1	7.9	0.01	0.11	0.72	1.16	0.06	0.09
	Max	4.6	8.8	0.06	0.12	0.93	1.31	0.07	0.12
	Mean	4.4	8.4	0.04	0.12	0.84	1.27	0.07	0.10
	St. Dev.	0.1	0.3	0.02	0.002	0.07	0.04	0.01	0.01
CM	Min	0.5	1.0	0.02	0.02	0.18	1.19	0.004	0.06
	Max	0.8	1.7	0.12	0.02	0.23	1.35	0.01	0.09
	Mean	0.6	1.3	0.07	0.02	0.20	1.27	0.01	0.07
	St. Dev.	0.2	0.2	0.03	–	0.01	0.06	0.003	0.01

nd: non detectable

The original article has been corrected.

